# Review of toxoplasmosis in Morocco: seroprevalence and risk factors for toxoplasma infection among pregnant women and HIV- infected patients

**DOI:** 10.11604/pamj.2017.27.269.11822

**Published:** 2017-08-10

**Authors:** Majda Laboudi

**Affiliations:** 1 Department of Parasitology, National Institute of Hygiene, 27 Avenue Ibn Batouta BP: 769, Agdal, Rabat, Morocco

**Keywords:** Toxoplasmosis, seroprevalence, risk factors, pregnant women, HIV-infected adults, Morocco

## Abstract

Toxoplasmosis is a disease caused by a protozoal parasite: *Toxoplasma gondii*. This infection can cause severe illness when the organism is contracted congenitally or when it is reactivated in immunosuppressed people. In this paper we review for the first time prevalence and risk factors of *T. gondii* among pregnant women and HIV-infected adults in Morocco. A systematic review methodology was used to consult three databases: Pub Med, Science Direct and Google Scholar dated until 2015, regarding prevalence data and risk factors of infection among pregnant women and people living with HIV. Data collection and eligibility criteria were established in this paper. No statistical method was employed in this study. Our review resulted in a total of 6 publications meeting the inclusion criteria of prevalence and risk factors of toxoplasmosis in Morocco. Seropositive rates of *T. gondii* infection reach up to 51% in pregnant women. Risk factors that were reported included contact with soil, lack of knowledge about toxoplasmosis, and a low educational level. For HIV-infected adults, the limited data show a 62.1% prevalence rate of *T. gondii* .According to our review, there is still very little information on toxoplasmosis disease in pregnant women and HIV infected patients in Morocco. Further research on toxoplasmosis is needed to better ascertain the human disease burden in Morocco.

## Introduction

Toxoplasmosis is a disease caused by intracellular protozoan parasite apicomplexan of worldwide distribution named *Toxoplasma gondii* [1, 2]. *T. gondii* can infect humans as well as virtually all warm-blooded animals, including mammals and birds [3]. Humans acquire the parasite by the oral route through the ingestion of cysts in the tissue of undercooked or uncooked meat, vegetables and fruits, or water contaminated with oocysts from infected cat feces [4–6]. Other means of transmission are organ transplantation [7], blood transfusion [8] and congenital transmission [4]. Toxoplasmosis has a cosmopolitan distribution: about one-third of the world”s population is infected with latent toxoplasmosis [3]. Approximately 30% of the human population worldwide is chronically infected with *T. gondii* [9, 10]. The incidence of toxoplasmosis differs, with underdeveloped countries having a higher incidence than developed countries. The highest prevalence is found in Latin America, parts of Eastern/Central Europe, the Middle East, and parts of South-East Asia and Africa [11]. The wide differences of seroprevalence depends on culture, eating habits [12] and climatic variations. This latter factor has a significant influence on the presence and persistence of infective oocysts, especially in tropical conditions where temperature, precipitation and humidity maintain higher soil moisture levels, allowing oocysts to persist and remain viable in the environment [10]. Although the toxoplasmosis infection is asymptomatic among immunocompetent individuals, it can lead to serious pathological effects in both immunodeficient patients and congenital cases [13]. When maternal infection is acquired during pregnancy, toxoplasma can infect the fetus with variable severity, depending on which trimester a pregnant woman is exposed to infection and on the efficacy of the placental barrier. The risk of congenital infection is relatively lower during the first trimester (10-15%) and highest when the infection occurs during the third trimester (60-90%). However, congenital infection during the first trimester can lead to more severe disease when it occurs [14]. The global annual incidence of congenital toxoplasmosis has been estimated to be 190,100 cases. Its equivalent to a burden of 1.20 million Disability Adjusted Life Years (DALYs) (95% CI: 0.76-1.90). The highest incidence rates occur in South America and in some Middle Eastern and low-income countries [15].

Furthermore, toxoplasmosis has emerged as a major opportunistic disease in patients with acquired immunodeficiency syndrome (AIDS). It can manifest as potentially fatal encephalitis, due to the reactivation of latent infections in HIV associated immune suppression [16, 17]. Toxoplasmosis ranks high on the list of diseases which lead to death in patients with AIDS; approximately 10% of AIDS patients in the USA and up to 30% in Europe are estimated to die from toxoplasmosis [4]. Toxoplasmosis is also a clinically important opportunistic infection in other immunosuppressed individuals such as patients who have had an organ transplant or are undergoing cancer treatment.

In Morocco, serological screening during pregnancy for toxoplasmosis is still not required by doctors. From 2006, decree 2519-05; 30 *Chaabane 1426 (BO no 5384 of 05 January 2006*) from the National Health Ministry of Morocco recommended, without obligation, the systematic serological screening of toxoplasmosis for pregnant women. The impact of toxoplasmosis on the health of mother and newborn should not be neglected. The surveillance of toxoplasmosis is mainly based on detection of antibodies IgG and IgM *T. gondii* [18]. The screening of the infection must be done early during pregnancy in order to facilitate interpretation of serological tests regarding the time of infection during pregnancy. Seroconversion, defined as appearance of IgG antibodies to *T. gondii*, will be detected by follow-up of serology in seronegative women during pregnancy. The aim of this paper is to review for the first time the published literature about the current status of relevant epidemiological aspects of *T. gondii* infection in pregnant women and immunosuppressed patients from Morocco.

## Methods

### Study area

Morocco is located at the northwestern corner of Africa. The area of the country is about 710,850 km^2^. Morocco is bordered to the west by the Atlantic Ocean, to the north by the Mediterranean Sea and is separated from Spain by the 14 km of the Strait of Gibraltar. It is also bordered to the East by Algeria and Mauritania to the South. Morocco is a country with an arid, semi-arid climate in the major part of the territory. The average annual precipitation varies from less than 100 mm (Saharan bioclimate) to 1200 mm (wet bioclimate). The main rivers flowing into the Atlantic are the Sebou and Oum Errabia. Four major mountain ranges are located in Morocco. They are wide-ranging throughout Morocco: the Rif, the Middle Atlas, High Atlas and Anti-Atlas. The country´s population in 2012 reached 32 million inhabitants, of which more than half live in urban areas. Morocco has: a monetary poverty rate of 9% (15% in rural areas, and 5% in urban areas) [19]; illiteracy rate of 43%, but higher (55%) among women; annual population growth rate of 1% in 2013 and total fertility rate (TFR) averaging two children per family; life expectancy of 75 years (77 yrs. in urban areas and 72 years. in rural areas); and a maternal mortality rate of 112 per 100,000 live births (report of *Ministry of health of Morocco, 2012*).

### Database search

**Figure 1:**
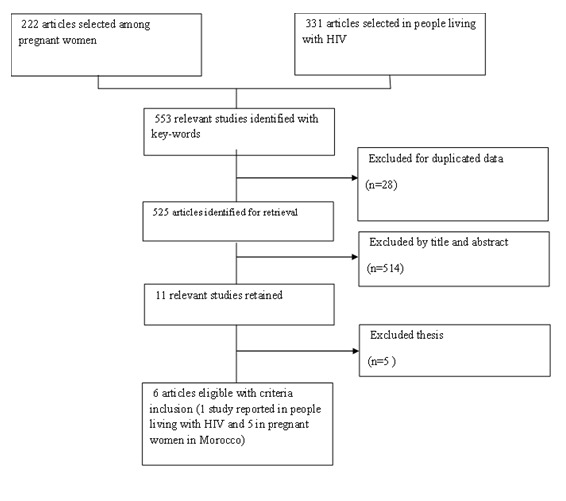
Flow chart for process of identification and selection of relevant studies (1983-2015)

In this review, we performed a systematic search of published papers reported from three databases (PubMed, Science Direct and Google Scholar) from 1983 to 2015 using the following research keywords including: “pregnancy OR pregnant women”, “prevalence OR seroprevalence”, “risk factors”, This was then followed by another search using the keywords HIV infected adults OR people living with HIV”. We combined the above words with “Toxoplasmosis OR *Toxoplasma gondii* infection in Morocco”. Criteria for inclusion were: the full text of papers written in English or French of studies carried out on pregnant women or HIV-infected patients in Morocco. Studies classified as citation, dissertation or thesis were excluded. Individual case studies were excluded also. No statistical method was employed in this study. Overall, 553 articles were discovered on the database between 1983 and 2015 using the key-words from which 28 duplicate article were removed. The titles and abstracts (525) were filtered using the criteria for inclusion and exclusion mentioned above. In the end, only 6 articles met criteria to be selected as eligible papers for this review (Figure 1). The extracted data included: year of publication, characteristics of the study population, location of the study, sample size, number of cases and diagnostic tests. These are summarized in the Table 1.

**Table 1:** Summary of *Toxoplasma gondii* prevalence in pregnant women and HIV infected people from Morocco

Years	Population surveyed	Location of study	Number of patients tested	Serological test	Prevalence(%)	Outcome measured	Reference
*1984*	*Pregnant women*	Casablanca	200	Not specified	51,5%	*Prevalence*	[19]
*2007*		Rabat	2456	ELISA	50.6%	*Prevalence*	[20]
*2009*		Rabat	1020	ELISA	51%	*Prevalence and Riskfactors*	[23]
*2010*		Rabat	500	ELISA	55 %+ 38,6 %++	*Prevalence*	[21]
*2014*		Rabat	1169	ELISA	47%	*Prevalence*	[22]
*2012*	*HIV positive adulte*	Marrakech	99	ELISA	62%	*Prevalence*	[24]

+ rural

++ urbain

## Current status of knowledge

### Toxoplasmosis among pregnant women in Morocco

#### Seroprevalence of Toxoplasmosis

Our articles included in our literature review describe the prevalence of toxoplasmosis among pregnant women in Morocco (Table 1). Early reports by Guessous et al, 1984 in Casablanca, a prospective serological study of 200 pregnant women, revealed that 51.5% are immune to toxoplasmosis. A study of the relation between the immune status and age shows that serum conversion takes place most frequently between 21 and 25 years old. The authors recommended that the prevention of congenital toxoplasmosis must be integrated into a national program of mother-and-child protection, notably by means of obligatory prenatal serological tests and continued monitoring of those women who are not immune to toxoplasmosis [20]. Another survey in Rabat (capital of Morocco) conducted by El Mansouri et al in 2007 found that among 2456 pregnant women sampled between 2002 to 2006, 51% had *T. gondii* antibodies. The seroprevalence of toxoplasmosis in pregnant women was estimated by using an ELISA test (IgG, IgM). Moreover, the use of the IgG avidity test had excluded a recent infection in 93.5% of pregnant women with positive IgM [21]. A prospective survey reported by Barakat et al in 2010, five hundred pregnant women, who had no serologic tests during pregnancy, were evaluated for *T. gondii* in the delivery room in a public hospital in Rabat. Among them, 55% of women from rural areas and only 38.6% from the urban areas were positive for antibodies to toxoplasma [22]. Additionally, one retrospective study by Laboudi et al in 2014 on the seroprevalence of toxoplasmosis among pregnant women assessed the role of parity, age and the presence or absence of abortion in the acquisition of the infection. The study showed that from 2008 to 2009 in Rabat, among 1169 pregnant women of different ages, 47% were found to be IgG seropositive, including 1.5 % IgM seropositive. The use of the IgG avidity test allowed for the exclusion of recent infection among 72.2 % of IgM positive sera. Detection of IgM was not always considered sufficient evidence of recent infection [23]. However, the contribution of the avidity test appears to be effective and reliable, allowing the exclusion of active toxoplasmosis [24].

A recent study in the Rabat region showed that the avidity test is a helpful tool to exclude a recently acquired toxoplasmosis infection within IgM-positive serum samples in pregnant women during their first trimester of pregnancy [25]. Throughout this survery, The authors et al report that the prevalence of IgG antibodies T. *gondii* increases with age in Morocco [18, 26]. The result of bivariate analysis revealed that age and parity significantly influenced the seroprevalence rate, however, the existence of previous spontaneous abortion did not have any significant statistical correlation with the positivity of toxoplasmosis [18]. Therefore, the prevalence increases with the number of pregnancies regardless of age. This study showed that 53 % of pregnant women were susceptible to T. *gondii* and considered to be at high risk for toxoplasmosis during pregnancy. The percentage of infected patients was highest among women aged over 40 years. These results were advanced by Berger et al in France who reported that toxoplasmosis infection increases linearly with age [27]. This can be explained by the increase in exposure to infection sources throughout life. On the other hand, one report recorded that there was no significant association of T. gondii infection with the existence of a history of spontaneous abortion [18]. In contrast, it was statistically significant with parity. Nevertheless, Breurec et al. (2004) reported that parity was not a significant factor. This is probably related to age because multiparous women are generally older than nulliparous women [1]. From our review, it's clearly seroprevalence of toxoplasmosis among pregnant women is quite high, with regional rates of seropositivity reach up to 51%. It appears that the prevalence in Morocco did not vary greatly from that previously found among pregnant women from neighboring Maghreb countries such as Algeria 47,8% [28] and Tunisia (47,7%) [29] as we share the same cultural and religious habits. In general, Toxoplasmosis is quite prevalent with regional rates of seropositivity upwards of 50% in Morocco while it's slightly lower suggesting that the variation of the prevalence can led to increase in seronegative pregnant women who are at high risk of developing congenital toxoplasmosis infection. Unfortunately, no study about the prevalence of congenital toxoplasmosis (CT) has been published in Morocco. Follow-up during pregnancy and pregnant women's awareness of the disease, remain essential to the prevention of congenital toxoplasmosis.

#### Risk factors among pregnant women

Only one survey was conducted by Laboudi et al, 2009 on the risk factors of toxoplasmosis in pregnant women in Rabat, Morocco. Another survey in Rabat (capital of Morocco) conducted by El Mansouri et al in 2007 found that among 2456 pregnant women sampled between 2002 to 2006, 50,6% had T. gondii antibodies. However, no statistical difference was found between raw meat consumption, possession of a cat and toxoplasmosis infection [26].In neighboring countries like Algeria, major risk factors were consumption of poorly-cooked meat and exposure to cats [28]. In Tunisia, the seropositivity for toxoplasmosis was significantly associated with eating undercooked meat and eating inadequately washed vegetables. However, the other factors (contact with cats, cleaning the cat litter box, washing the hands after preparation of raw meat, contact with the ground) were found to be independently associated with seropositivity toxoplasmosis [29]. Regarding occurrence in Europe, Toxoplasma infection can be explained by changes in risk factors attributed to various causes related to undercooked meat [30]. In Central America and in other developed countries, toxoplasmosis prevalence may be related to socioeconomic status and the presence of stray cats, especially in a climate suitable for the survival of oocysts.

### Toxoplasmosis among people who live with HIV

Even though the prevalence of HIV is low (0.15%) in Morocco [31], the only report by Abbdous et al in 2011showed a high prevalence of *T. gondii* in these patients (62%) [32]. Therefore, HIV-infected patients in the Marrakech region could be at high risk of developing toxoplasmosis disease, especially when the CD4+ T-cells count falls below 100 cells/l. In this only study, *Toxoplasma* seropositivity was not influenced by age, gender, ART status, or rural/urban area. Also, there was no significant difference between a mean of CD4+ T-cells count of the positive serology group (378.8 ± 215.1 cells/l) and the negative serology group (394.3 ± 274.2 cells/l) [32]. The authors of this study highlights the importance of monitoring patients with HIV antibodies due to the high risk of cerebral toxoplasmosis in HIV-infected patients with CD4+ cells below 100 cells/μl. On the other hand, the 37.9% of patients, who have had no prior contact with *T. gondii* , need health education about the transmission modes of toxoplasmosis and hygiene rules to prevent contamination. Accordingly, there is a serious need for widening antiretroviral therapy and chemoprophylaxis against toxoplasmosis, when indicated, to avoid toxoplasmosis reactivation among this population.

## Conclusion

According to our review, there is no recent published study concerning the prevalence of toxoplasmosis within the general population in our country, as all the studies thus far come only from the Rabat, Casablanca and Marrakech regions. There is a lack of relevant studies in many areas of the country. Furthermore, no recent study exists on the prevalence of congenital toxoplasmosis in Morocco. On the other hand, the variation of the prevalence of toxoplasmosis during recent years suggest an increase in seronegative pregnant women who are at high risk of developing clinical signs associated with the toxoplasma infection. It is necessary to inform currently unexposed pregnant women and HIV infected patients about the importance of the disease and the impact of the toxoplasma infection. Furthermore, washing hands before and after handling food may play a role in reducing the risk of contamination of uninfected women. Also, the use of gloves when changing cat litter, gardening and other contacts with the soil could remove the risk of the disease occurring during the period of pregnancy. This can only be possible if these preventive tips are integrated with school health activities and health education campaigns. Obviously, implementation of regulation and surveillance programs for the prevention and control of toxoplasmosis in Morocco should be considered. Additionally, encouraging research on toxoplasmosis in Morocco will help assess the real burden of this disease in humans and especially in pregnant women and congenitally infected children.

### What is known about this topic

- The importance of Toxoplasmosis disease in the world;
- This infection can cause severe illness when the organism is contracted congenitally or when it is reactivated in immunosuppressed people;
- The absence of the program of Toxoplasmosis disease in Morocco.

### What this study adds

- The limited data about prevalence and risk factors of Toxoplasmosis disease in Morocco among pregnant women;
- The limited data about prevalence of Toxoplasmosis disease in Morocco among HIV patients;
- The program of Toxoplasmosis disease in Morocco remains challenging.

## Competing interests

The author declares no competing interests.
